# Polymer-Based Graphene Derivatives and Microwave-Assisted Silver Nanoparticles Decoration as a Potential Antibacterial Agent

**DOI:** 10.3390/nano10112269

**Published:** 2020-11-16

**Authors:** Angelo Nicosia, Fabiana Vento, Anna Lucia Pellegrino, Vaclav Ranc, Anna Piperno, Antonino Mazzaglia, Placido Mineo

**Affiliations:** 1 Department of Chemical Sciences, University of Catania, V.le A. Doria 6, 95125 Catania, Italy; fabiana.vento@phd.unict.it (F.V.); annalucia.pellegrino@unict.it (A.L.P.); 2 Regional Centre of Advanced Technologies and Materials, Palacký University Olomouc, Šlechtitelů 11, 78371 Olomouc, Czech Republic; vaclav.ranc@upol.cz; 3 Department of Chemical, Biological, Pharmaceutical and Environmental Sciences, University of Messina, V.le F. Stagno d’Alcontres 31, 98166 Messina, Italy; apiperno@unime.it; 4 CNR-ISMN, Istituto per lo Studio dei Materiali Nanostrutturati, V. le F. Stagno d’Alcontres 31, 98166 Messina, Italy; antonino.mazzaglia@cnr.it; 5 Institute for Chemical and Physical Processes CNR-IPCF, Viale F. Stagno d’Alcontres 37, 98158 Messina, Italy; 6 Institute of Polymers, Composites and Biomaterials CNR-IPCB, Via P. Gaifami 18, 95126 Catania, Italy

**Keywords:** NanoHy-GPS, antibacterial nanosystems, one-pot microwave-assisted reaction, graphene oxide, silver nanoparticles, polyvinyl alcohol

## Abstract

Nanocomposites obtained by the decoration of graphene-based materials with silver nanoparticles (AgNPs) have received increasing attention owing to their antimicrobial activity. However, the complex synthetic methods for their preparation have limited practical applications. This study aims to synthesize novel NanoHybrid Systems based on graphene, polymer, and AgNPs (namely, NanoHy-GPS) through an easy microwave irradiation approach free of reductants and surfactants. The polymer plays a crucial role, as it assures the coating layer/substrate compatibility making the platform easily adaptable for a specific substrate. AgNPs’ loading (from 5% to 87%) can be tuned by the amount of Silver salt used during the microwave-assisted reaction, obtaining spherical AgNPs with average sizes of 5–12 nm homogeneously distributed on a polymer-graphene nanosystem. Interestingly, microwave irradiation partially restored the graphene sp^2^ network without damage of ester bonds. The structure, morphology, and chemical composition of NanoHy-GPS and its subunits were characterized by means of UV-vis spectroscopy, thermal analysis, differential light scattering (DLS), Field Emission Scanning Electron Microscopy (FE-SEM), Energy Dispersive X-ray analysis (EDX), Atomic Force Microscopy (AFM), and High-Resolution Transmission Electron Microscopy (HRTEM) techniques. A preliminary qualitative empirical assay against the typical bacterial load on common hand-contacted surfaces has been performed to assess the antibacterial properties of NanoHy-GPS, evidencing a significative reduction of bacterial colonies spreading.

## 1. Introduction

The properties of the polymer-based materials have led to their ubiquitous application as structural material not only for common-use objects but also for value-added devices. As examples, these materials are employed in manufacturing of kids’ toys, but also to produce biomedical devices such as catheters, ureteral stents, and prosthesis. Especially in the biomedical field, severe infections could occur using invasive devices, due to bacterial contaminations. Besides the necessity to sterilize the materials before their use [1], antimicrobial agents are needed to provide long-term antibacterial efficacy [2]. With this aim, low-weight organic molecules are usually applied as antimicrobial agents and used through spray coating techniques or blended into the polymer matrix during its processing [3,4].

If any bactericidal agent is applied onto the surface of a material, the bacterial adhesion [5] comes in succession due to the reproduction and the formation of colonies, which develop in biofilms (a secretion of exopolysaccharides) [6,7], a protective agent against bactericidal and bacteriostatic substances [8,9,10,11,12].

Viruses also could take advantages from the biofilm, exploiting it as a shield from the environmental stresses, allowing the contamination through biofilm spreading [13]. Such an occurrence represents a huge issue, especially in the latest Severe Acute Respiratory Syndrome CoronaVirus-2 (SARS-CoV-2) pandemic context. To limit the contagion possibilities, the demand of redox-based disinfectant agents to sanitize surfaces has seen an exponential rise, resulting in toxic side-effects towards the environment and wildlife [14].

Moreover, the low adhesiveness, the ubiquitous use of organic-based antibacterial agents, and the subsequential release into the environment has caused direct exposure to life forms, resulting in bioaccumulation for several species worldwide, including humans [15,16], and acting as endocrine disruptors [17]. For these reasons, some of these molecules have also been banned from both European and American health institutions [18]. Nevertheless, the ban concerned only some application fields—these additives are still used, especially as biomedical devices coatings (i.e., surgical suture wires), because of their efficiency towards multiple bacterial targets [18].

An alternative approach to overtaking such a huge issue is represented by surfaces and/or materials having intrinsic long-term antibacterial properties.

In this landscape, a potential solution could be antibacterial polymer-based coatings [19]. Polymers can assure all the required features such as easiness of synthesis and application, long-term stability in environmental conditions, and absence of any degradation product or toxic product leaking. Moreover, antimicrobial agents [19,20] could be loaded in the polymer matrix, performing their long-term activity towards pathogens.

In the field of nanotechnology, Silver NanoParticles (AgNPs) exhibit a broad-spectrum antibacterial activity, against Gram-positive and Gram-negative micro-organisms [21,22,23,24], and also multidrug-resistant bacteria [25]. The antibacterial efficiency is attributed to a multifaceted mechanism lying on the release of silver ions [26,27,28,29]. The continuous increase of market products containing silver nanoparticles raises the issue of the risks associated with their release in the environment and on the consequent negative effects on human health [30,31,32].

Graphene Oxide (GO) is a versatile material made up of mono- or few-layers carbon honeycomb structure functionalized with oxidized species (alcohols, epoxides, carboxylic acids), proposed in many application fields. Engineered GO-based materials, due to their biocompatibility, were also proposed as drug delivery systems and for nanomedicine applications [33,34,35,36].

Controversial literature data have been reported about the antimicrobial properties of GO; however, its dual oxidative and membrane stress effect has been proven [37,38,39,40]. It was verified that polymer coatings containing a suitable amount of GO could prevent the metal substrate from oxidation and bacterial adhesion, while maintaining a positive cell adhesion and response exploitable for surgery implants [41].

The unique features of graphene [37,39] and the antimicrobial properties of the AgNPs could be merged into a complex hybrid system, showing enhanced synergistic antimicrobial effect than the single moiety or their blend [40,42,43]. Moreover, the anchoring of AgNPs onto the graphene platform reduces the risk of aggregation [44,45,46].

In this framework, our interest has been addressed on the development of a fine-tuned hybrid system combining the properties of polymer, graphene, and AgNPs as a potential on-demand antimicrobial multisurface coating system.

In this system, polymer plays a crucial role since it assures the coating layer/substrate compatibility, making the platform easily adaptable for a specific substrate by changing the polymer of the hybrid system.

In order to ensure the interchangeability of the polymer moiety, the GO functionalization was performed by a synthetic strategy suitable for several polymers. Here, PolyVinyl Alcohol (PVA) was selected as a model moiety and used to produce a PVA@GO covalent system.

Finally, employing a microwave-assisted method to perform a one-pot reaction concerning the simultaneous reduction of AgNPs and GO, nanohybrid systems were obtained (here called PVA@rGO-AgX). The structure, morphology, and chemical composition of PVA@rGO-AgX and its subunits were characterized by means of UV-vis spectroscopy, thermal analysis, differential light scattering (DLS), Field Emission Scanning Electron Microscopy (FE-SEM), Energy Dispersive X-ray analysis (EDX), Atomic Force Microscopy (AFM), and High-Resolution Transmission Electron Microscopy (HRTEM) techniques. Finally, preliminary biological tests have been performed as a proof of concept for its antibacterial activity.

The synthetic pathway exposed here allows fine-tuning of the nanosystem features, thus representing the first approach towards the synthesis of a novel class of NanoHybrid Systems based on Graphene, Polymer, and Silver, namely, NanoHy-GPS.

To our knowledge, an on-demand polymer antibacterial coating nanosystem has not been found yet.

## 2. Materials and Methods

### 2.1. Synthesis

Natural graphite powder (diameter 5–10 μm, thickness 4–20 nm, layers < 30, purity > 99.5 wt.%), polyvinyl alcohol, silver nitrate, and all the other reagents and solvents used in this work were purchased by Sigma-Aldrich (Merck Group, Milan, Italy).

#### 2.1.1. GO Synthesis

Graphene oxide (GO) was prepared by oxidizing graphite powders (diameter 5–10 μm, thickness 4–20 nm, layers < 30) according to our previously reported procedure [34] using the Hummers method [47]. Briefly, graphite (2 g) and concentrated sulfuric acid (350 mL) mixture was cooled at 0 °C under stirring. Then, sodium nitrate (1 g) and potassium permanganate (8 g) were slowly added, the temperature was raised up to 40 °C, and the mixture stirred for 1 h. Deionized water (250 mL) was slowly added into the solution (determining an increase of temperature up to 70 °C), the temperature was raised up to 98 °C, and the mixture was stirred for 30 min. Finally, 52 mL of H_2_O_2_ (30%) was added and the bright yellow suspension was filtered by using a Millipore Membrane (0.1 μm) under vacuum and washed with HCl (4%) and water to reach a neutral pH. The solid was dried to obtain the graphite oxide as a brown powder (1.8 g). Aqueous suspension of graphite oxide (500 mg in 35 mL of water) was exfoliated by ultrasonication (40% W, 8 h) using a UW 2070 SONOPLUS, Bandelin Electronic (Berlin, Germany). The dispersion was diluted with deionized water and centrifugated (10,000 rpm for 12 min). The GO supernatant was dried to recovery GO powder.

#### 2.1.2. Polymer-GO Covalent Adduct Synthesis

The Graphene-Oxide–Polyvinyl-alcohol esterification (PVA@GO) was conducted through a slightly modified method developed by Salavagione et al. [48]. Briefly, 20 mg of GO and 200 mg of PolyVinyl Alcohol (PVA) were dissolved in dimethyl sulfoxide (DMSO, 10 mL) at 70 °C under stirring in nitrogen atmosphere. After 24 h, the mixture was cooled at room temperature. Then, *N*,*N*′-Dicyclohexylcarbodiimide (DCC, 926 mg, 4.5 mmol) and *N*,*N*-Dimethylpyridin-4-amine (DMAP, 69 mg, 0.56 mmol), previously solubilized in DMSO (10 mL) in nitrogen atmosphere, were added to the PVA solution. The reaction was kept under stirring in nitrogen atmosphere for 3 days; after that, the mixture was precipitated in methanol (50 mL) and centrifuged (9000 rpm, 20 min). The precipitate was dispersed in 70 mL hot water (70 °C). So, the water solution, containing PVA@GO, was concentrated by means of a rotavapor, and then coagulated in methanol and centrifugated (9000 rpm, 20 min). The procedure was repeated three times. The precipitate was dried for 24 h in oven (50 °C) under vacuum.

#### 2.1.3. Microwave-Assisted Silver Nanoparticles Synthesis

In order to reduce the silver nanoparticles on the GO platform, a microwave-assisted reaction was conducted on PVA@GO aqueous solution (0.5 mg/mL). The solution was put in a 10 mL vessel and proper amounts of Silver Nitrate (AgNO_3_) were added. Once the solution was stirred, a suitable amount (5 mL) of Dimethylformamide (DMF) was added and the mixture was sonicated for 2 min. The vessel was capped and inserted into the microwave holder. The reaction was conducted at a fixed power of 300 W for two minutes, cooling the system by means of air flux.

The total reaction mixture was mixed with methanol (20 mL) and concentrated using a rotary evaporator (60 °C and vacuum). Then, the mixture was precipitated in ethyl ether (25 mL), the solid was separated through centrifugation (9000 rpm, 20 min), then dried in a vacuum oven at 50 °C overnight.

By increasing the AgNO_3_ amount (35.7 μg (0.21 μmol), 0.33 mg (1.96 μmol), and 3 mg (18.7 μmol)) added during the synthetic procedure, three products were obtained, namely, PVA@rGO-Ag1, PVA@rGO-Ag2, and PVA@rGO-Ag3, respectively.

For sake of comparison, by means of the same microwave-assisted procedure, without using the AgNO_3_, samples of reduced PVA@GO (called PVA@rGO) and a sample of reduced GO (called rGO) were produced as well.

#### 2.1.4. Sodium-Borohydride-Mediated Silver Nanoparticles Synthesis

Besides, Silver Nanoparticles (AgNPs) have been also synthetized through the typical chemical reduction of silver nitrate in aqueous solution [49]. Briefly, 750 mL of sodium borohydride water solution (2 mM) were prepared and left under high-speed stirring. Then, 250 mL of silver nitrate water solution (1 mM) was added through a dropping funnel. The reaction was left under high stirring until the end of the silver nitrate solution. In order to stabilize the suspension and have certainty about the reduction of the whole silver salt added, the mixture was stirred for an additional 30 min. The obtained AgNPs suspension, stored at 5 °C, is stable for months. The Localized Surface Plasmon Resonance (LSPR) signal at 394 nm of such AgNPs has been checked by UV-vis measurements.

#### 2.1.5. Preliminary Antibacterial Tests

Bacterial population from common hand-contacted surfaces have been recovered and streaked [50] over the Plate Count Agar (PCA). In detail, sterile swabs, premoistened in Maximum Recovery Diluent (MRD), were streaked over common hand-contacted surfaces (i.e., door handles and handrails) with size 100 cm^2^. In order to ensure a good capture of bacteria, the swab constantly rotated and uniformly swiped in all directions of the tested surface. Then, the microorganisms transferred to the swab were released into 10 mL of MRD, transported in the microbiology laboratory (about 5 min), and used to test the several compounds, namely, AgNPs, PVA@rGO-Ag2, PVA@rGO-Ag1, AgNO_3_, and PVA@GO. Each compound (100 μL, 27 μg/mL in Ag content and/or 142 μg/mL in PVA@GO content) was previously deposited onto the PCA. Then, bacteria in MRD (100 μL) were spread on treated PCA and incubated for 24 h at 37 °C. Each biological experiment was performed in triplicate. As control, bacteria in MRD without any compound was used. Microbial growth was evaluated according to the Colony-Forming Unit (CFU) assay, assuming that each colony has originated from a single bacterium [51]. The CFU for cm^2^ of sampling surface (CFU/cm^2^) value were calculated from Equation (1):

CFU/cm^2^ = (Number of Colonies × Volume of MRD (10 mL))/(Volume spread on PCA (0.1 mL) × Sampled surface (100 cm^2^)). (1)

### 2.2. Methods

UV-vis spectra were recorded at 25 °C by a Cary 60 UV-vis spectrophotometer (Agilent Technologies, Santa Clara, CA, USA) in quartz cells (1 cm optical path), using water as a solvent. The concentration of the analyzed systems were 0.3 mg/mL for GO, rGO, PVA, PVA@GO, and PVA@rGO; 3.5 mg/L for AgNPs; instead, the PVA@rGO-Ag sample concentration was 30 μg/mL due to the high intensity of the LSPR signal.

^1^H NMR and COSY spectra (acquired at 27 °C with a spin lock time of 0.5 s) were obtained on a ^UNITY^INOVA instrument (Varian, Agilent Technologies, Santa Clara, CA, USA) operating at 500 MHz and using VNMR for acquisition and spectra processing. Samples were dissolved in water-d6 and the chemical shifts expressed in ppm by comparison with the water residue signal.

High-pressure microwave-assisted reactions were performed in a single-mode microwave reactor CEM Discover S-Class (CEM Corporation, Matthews, NC, USA) equipped with a calibrated infrared temperature sensor, employing capped sealed pressure-rated vessels (10 mL).

The Dynamic Light Scattering (DLS) measurements were performed by a miniDAWN Treos (Wyatt Technology, Santa Barbara, CA, USA) multiangle light scattering detector, equipped with a Wyatt QELS DLS Module. The measurements were performed at 25 °C using water (LC-MS grade) as a solvent, previously filtered with 0.2 μm filter. Size distributions were obtained using the ASTRA 6.0.1.10 software (Wyatt Technology, Santa Barbara, CA, USA).

Thermogravimetric analyses were performed by means of Pyris TGA7 (Perkin Elmer, Waltham, MA, USA) in the temperature range between 50 and 800 °C, under an air flow of 60 mL min^−1^ and heating rate of 10 °C min^−1^.

The combined system NTEGRA Spectra (NT-MDT Co., Zelenograd, 124482, Moscow, Russia) was utilized to acquire sample topography and Nova Px ver 3.4.0 rev. 19040 software (NT-MDT Co., Zelenograd, 124482, Moscow, Russia) was used for the data analysis. The surface morphology was obtained by the means of semicontact mode (height and phase) with the NSG30 (High Resolution NONCONTACT “GOLDEN”, NT-MDT, Moscow, Russia) cantilever having a force constant of 22–100 N/m and resonant frequency 240–440 kHz, and the ACTA-SS (AppNano, Mountain View, CA, USA) cantilever having a force constant of 13–77 N/m and resonant frequency of 200–400 kHz. The scanning rate was 0.3 Hz. During the measurement, the humidity was in range of 45–55% and the temperature was RT. The height profile was calculated using Gwyddion 2.51 software [52]. The sample was dissolved in water and sonicated for at least 60 min. Total volume of 3 μL of the as-prepared sample was deposited at freshly cleaved MICA substrate via drag and drop method and left to dry.

The morphology was investigated using the field emission scanning electron microscope ZEISS Supra 55 VP (Zeiss, Oberkochen, Germany). The atomic composition of the samples was analyzed through energy dispersive X-ray analysis, using an INCA-Oxford windowless detector, having a resolution of 127 eV at the full-width half-maximum (FWHM) of the Mn Kα.

HRTEM images were obtained using a high-resolution transmission electron microscope HR-TEM FEI Titan G2 60–300 (Thermo Fisher Scientific, Waltham, MA, USA) with an X-FEG type emission gun, operating at 80 kV. This microscope was also supplied with a Cs image corrector and a STEM High-Angle Annular Dark-Field detector (HAADF). For these analyses, a droplet of the material ultrapure H_2_O dispersion (0.1 mg mL^−1^) was deposited onto a carbon-coated copper grid and dried.

## 3. Results

### 3.1. Preparation of PVA@rGO-Ag Hybrid Systems

**Scheme 1:**
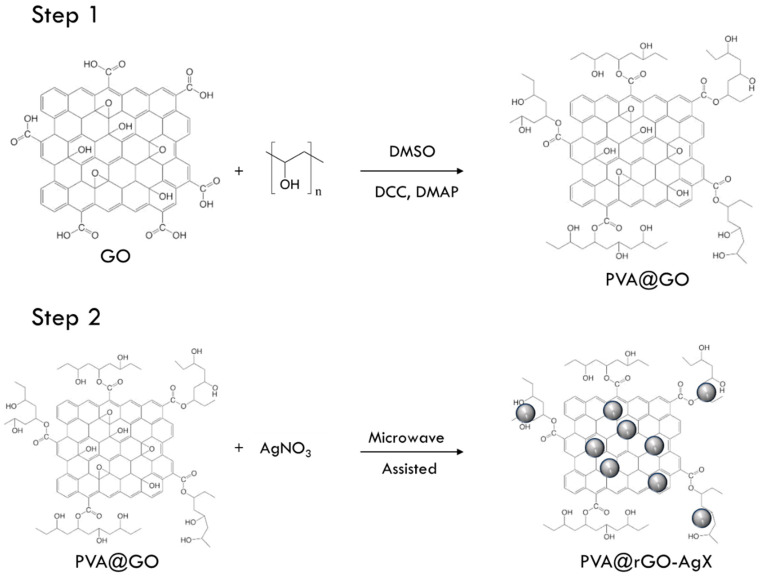
Schematic representation of PolyVinyl Alcohol-Graphene Oxide-silver hybrid systems (PVA@rGO-AgX) preparation.

The esterification of the GO was performed exploiting the PVA chains hydroxyl side-groups, as described in Scheme 1 (Step 1), using DCC and DMAP as coupling reagents. In order to promote the anchoring of AgNPs on GO platform, the direct reduction microwave-assisted reaction of silver ions was performed (Scheme 1, Step 2). PVA@GO and AgNO_3_ were well dispersed in water/DMF and subjected to microwave irradiation for 2 min (300 W, temperature below 180 °C under pressurized air cooling). PVA@rGO-Ag1, PVA@rGO-Ag2, and PVA@rGO-Ag3 samples were obtained increasing the AgNO_3_ amount (see experimental procedure). Samples called PVA@rGO (reduced PVA@GO) and rGO (reduced GO) were obtained by microwave irradiation of GO and PVA@GO and used to investigate the restoring of graphene sp^2^ network.

### 3.2. Characterization

The molecular structure of the GO polymer derivative (PVA@GO) was confirmed by ^1^H-NMR and COSY spectroscopies. The ^1^H NMR spectrum of the PVA@GO (Figure S1) shows the typical signals of PVA protons at 1.15–1.97 ppm (CH_2_), and at 3.57–4.17 ppm (CH). Noteworthily, the appearance of the novel signal at 5.05 ppm, attributed to the ester CH protons, proved the successful esterification reaction. The correlations between methylene protons (1.15–1.95 ppm) and CH protons at 5.05 ppm in the COSY-NMR spectrum (Figure S2) further confirms the occurrence of the esterification reaction with a degree of functionalization comparable with literature data (about 2.5%) [48].

A microwave-assisted reaction was set up to decorate the PVA@GO nanosystem with different amounts of AgNPs. The same procedure was applied to GO and PVA@GO in the absence of silver salt (see Experimental Section).

**Figure 1:**
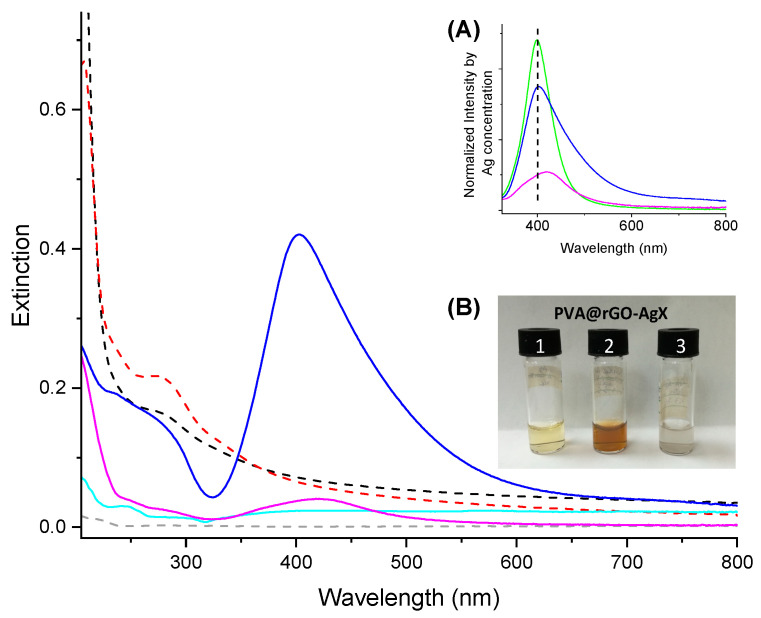
UV-vis spectra of water dispersions/solutions of PolyVinyl Alcohol (PVA) (grey dashed line, 0.3 mg/mL), PVA@GO (red dashed line, 0.3 mg/mL), PVA@rGO (black dashed line, 0.3 mg/mL), PVA@rGO-Ag1 (magenta line, 30 μg/mL), PVA@rGO-Ag2 (blue line, 30 μg/mL), PVA@rGO-Ag3 (cyan line, 30 μg/mL). Inset (**A**): UV-vis spectra normalized by Ag weight content of AgNPs (green line), PVA@rGO-Ag1 (magenta line), PVA@rGO-Ag2 (blue line). Inset (**B**): Digital image of water dispersions (0.3 mg/mL) of PVA@rGO-Ag1 (vial 1), PVA@rGO-Ag2 (vial 2), and PVA@rGO-Ag3 (vial 3).

The spectroscopic properties of the nanohybrid systems were investigated in water dispersion through UV-vis spectroscopy (Figure 1). The PVA spectrum (grey dashed line, 0.3 mg/mL) indicates a negligible absorption signal. On the other hand, PVA@GO (red dashed line, 0.3 mg/mL) exhibits a wide scattered signal from 200 nm to 800 nm.

It is noticeable that the microwave-assisted reaction determines the GO platform reduction of the PVA@GO, obtaining a new system named PVA@rGO (black dashed line, 0.3 mg/mL), as suggested by changes in the absorption curve slope.

To confirm this hypothesis, a sample of GO was treated in microwave-assisted reaction conditions. The red-shift of the π-π* transition peak from 237 nm (attributed to GO, Figure S3) to 266 nm (attributed to rGO, Figure S3), together with a different slope, confirmed that the reduction of GO occurred. The rGO shows also an absorption signal at 205 nm, according to the increase of π-π attraction between rGO platforms [53].

The Localized Surface Plasmon Resonance (LSPR) provides qualitative considerations about the AgNPs deposited onto the hybrid nanosystem.

As expected, UV-vis spectra of the three PVA@rGO-AgX water dispersion (30 μg/mL) show spectroscopic differences due to the different Ag content and to the interactions with the polymer-based substrate. In particular, the PVA@rGO-Ag1 (Figure 1, magenta line) shows the LSPR extinction signal at 420 nm (FWHM about 100 nm), while PVA@rGO-Ag2 (Figure 1, blue line) shows a band centered at 403 nm (FWHM about 100 nm), generally corresponding to AgNPs size of about 50 nm and 15 nm, respectively [54].

To shed light on the substrate influence towards the AgNPs formation, in Figure 1A, we report the UV-vis profile as normalized intensity by Ag concentration of free AgNPs (green line), PVA@rGO-Ag1 and -2 (magenta and blue line, respectively) vs. wavelength. In contrast with UV-vis of the free AgNPs spectrum (showing LSPR at 394 nm, FWHM about 60 nm), broadened and weaker LSPR bands are exhibited by both the PVA@rGO-AgX systems. This variation could be due to the interaction with the polymer-based substrate [55,56], as confirmed by the increase of LSPR red-shift by decreasing the Ag content into the nanohybrid systems.

The different color of the PVA@rGO-Ag1 and -2 water dispersions (respectively, brownish and yellowish; see Figure 1B) is a naked-eye evidence of their quali- quantitative differences.

In the case of PVA@rGO-Ag3, the higher amount of Ag salt in the synthesis procedure determines the formation of agglomerated broad-sized Ag particles, resulting in unstable microscopic suspension (Figure 1, cyan line, and Figure 1B).

**Figure 2:**
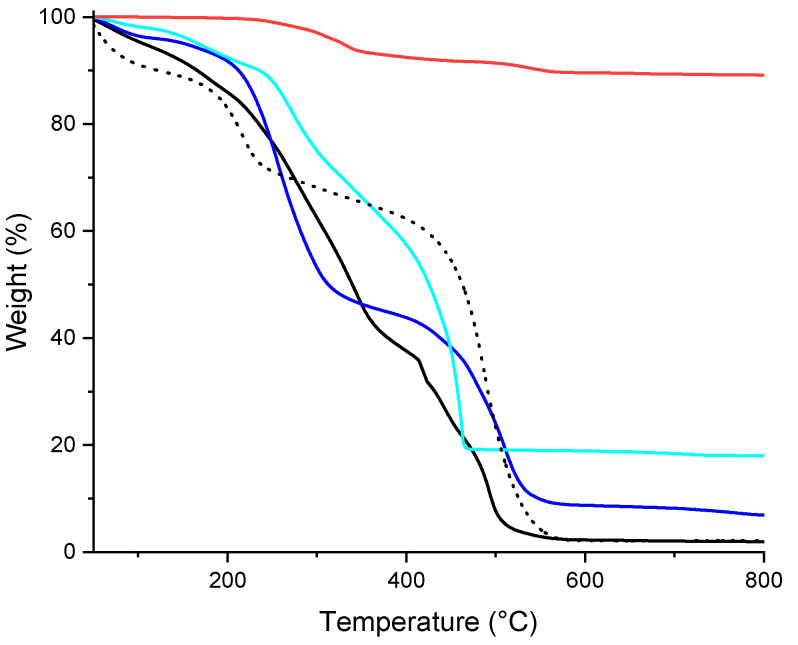
Thermogravimetric analysis (TGA) thermograms of Graphene Oxide (GO) (dotted line), PVA@GO (black line), PVA@rGO-Ag1 (blue line), PVA@rGO-Ag2 (cyan line), and PVA@rGO-Ag3 (red line), air atmosphere.

The quantitative determination of the Ag on the nanosystems was performed through thermogravimetric analysis (Figure 2) [46,57,58]. The Ag weight (%) content in the different samples has been estimated, obtaining 5%, 16%, and 87% (*w/w*) of Ag for PVA@rGO-Ag1, -2, and -3, respectively. For the sake of clarity, TGA traces of GO (dotted line) and PVA@GO (black line) are also reported.

**Figure 3:**
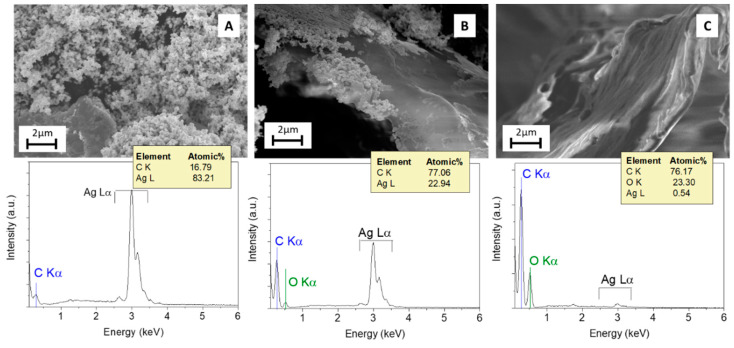
(**A**) Field Emission Scanning Electron Microscopy (FE-SEM) images and the related Energy Dispersive X-ray analysis (EDX) spectra of PVA@rGO-Ag3; (**B**) PVA@rGO-Ag2; (**C**) PVA@rGO-Ag1.

A morphological and qualitative characterization of the hybrid nanosystems has been conducted employing FE-SEM and EDX investigations (Figure 3). All the PVA@rGO-Ag nanohybrid systems present similar smooth surfaces attributed to PVA@rGO backbone. The AgNPs displayed spherical and quite regular morphology in all three analyzed samples, with a distribution of agglomerates in samples PVA@rGO-Ag2 (Figure 3B) and, in particular, a very large distribution in PVA@rGO-Ag3 (Figure 3A). The EDX analysis confirms the nature of the nanoparticles, as evidenced by a peak at 2.98 keV attributed to Ag Lα line, and shows a strong decrease of Ag atomic percentage from PVA@rGO-Ag3 to PVA@rGO-Ag1 of 83%, 22%, and 0.5% (Figure 3A–C), respectively. In addition, only the signal at 0.28 keV related to the C Kα peak arising from PVA@rGO backbone is present.

The PVA@rGO-Ag1 represents the most reliable sample to investigate the nanohybrid system morphology, because of the lack of AgNPs agglomerates. In fact, despite the absence of AgNPs evidence in the FE-SEM image, the related EDX spectra (Figure 3C) confirms the AgNPs presence, which are well-dispersed and embedded within the PVA@rGO backbone.

**Figure 4:**
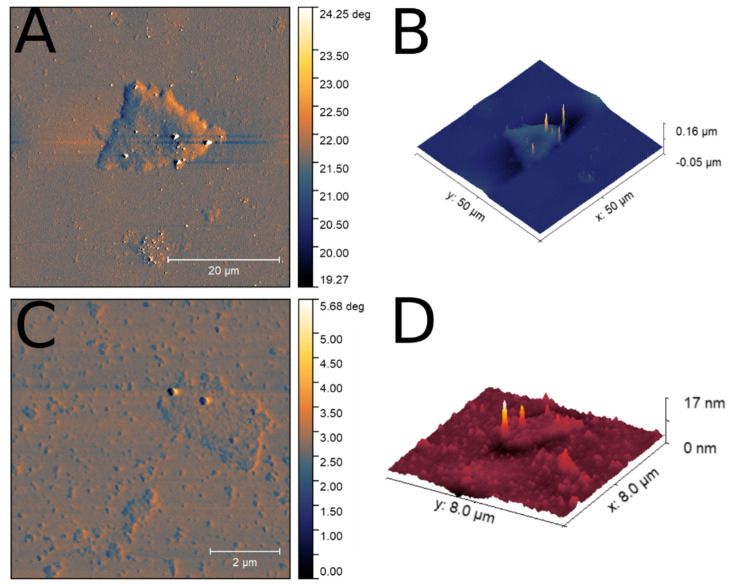
Atomic Force Microscopy (AFM) phase images of PVA@rGO-Ag1 (**A**) 50 × 50 µm and (**C**) 8 × 8 µm and (**B**,**D**) the related 3D Height.

PVA@rGO-Ag1 sample was characterized by means of HRTEM and AFM analyses. AFM was used to investigate the localization of Ag nanoparticles on the surface of rGO and to evaluate the topography of synthesized hybrid nanostructures. It is hypothesized that a considerably higher value of Ag concentration would indeed cover the rGO moiety (as evidenced by SEM analysis in Figure 3A). The phase images (Figure 4A,C and related 3D rendering in Figure 4B,D) confirm this and show that the sample is in the form of a PVA homogeneous film containing both AgNPs and rGO platform. The height profile calculations (shown in Figure S5) revealed that the rGO moiety is basically a few layers of aggregates with a height of around 50 nm. Its surface is functionalized by PVA and covered by AgNPs. According to the UV-vis results, the AgNPs size distribution ranged from few to dozens of nanometers.

**Figure 5:**
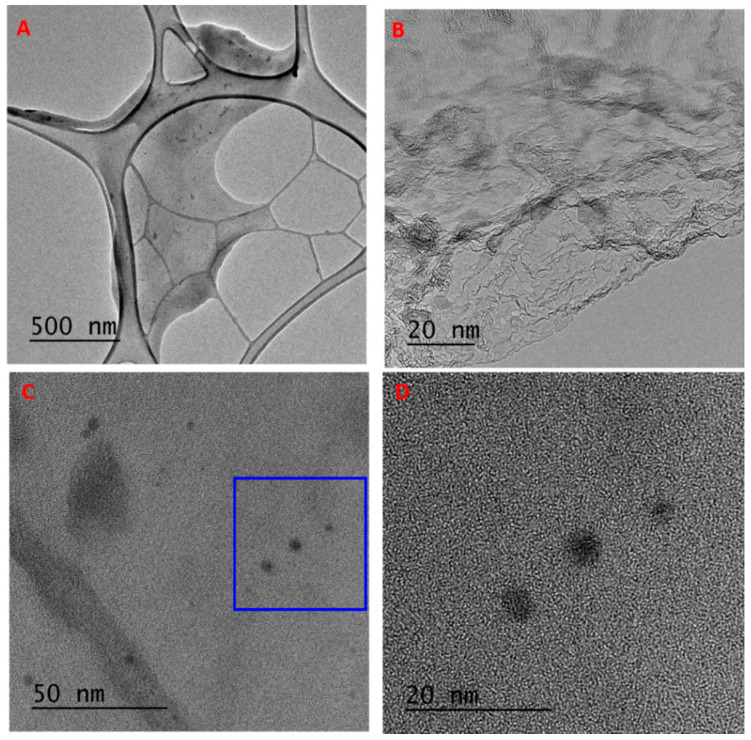
Representative high-resolution transmission electron microscopy (HRTEM) images of PVA@rGO-Ag1 (**A**–**D**). Area visualized in (**D**) is highlighted in (**C**) by the blue rectangle.

HRTEM images of PVA@rGO-Ag1 (Figure 5A) show the typical morphology of hybrid nanocomposite materials. Taking into account that the starting GO is produced as folded and large sheets, the effects of the concurrent microwave irradiation and deposition of AgNPs have promoted the unfolding and formation of well-exfoliated graphene sheets having different levels of transparency. As a result, PVA@rGO-Ag1 shows the typical morphology of functionalized reduced graphene (Figure 5B), as observed in our previous papers [34,35], evidencing planar backbone conformation and transparency. The AgNPs were detected as dark spots homogeneously dispersed over the PVA@rGO background material (Figure 5C,D), confirming a random formation of nucleation site within the polymer-based nanosystem. The AgNPs localization, also within the PVA matrix, is in accordance with literature data, since the PVA acts as a capping agent for AgNPs [59,60,61,62]. Thus, GO-oxygenated functional groups and PVA -OH groups act as homogeneous NPs stabilizers. Considering the AgNPs’ size obtained by HRTEM and UV-vis analyses, the data apparently do not match each other: this ostensible contradiction could be explained by considering the observable properties of the system. Indeed, while HRTEM technique reports the morphology of the system in dry state, the LSPR signal is influenced by the chemical environment of the AgNPs (solvent, PVA -OH groups, graphene, etc.).

**Figure 6:**
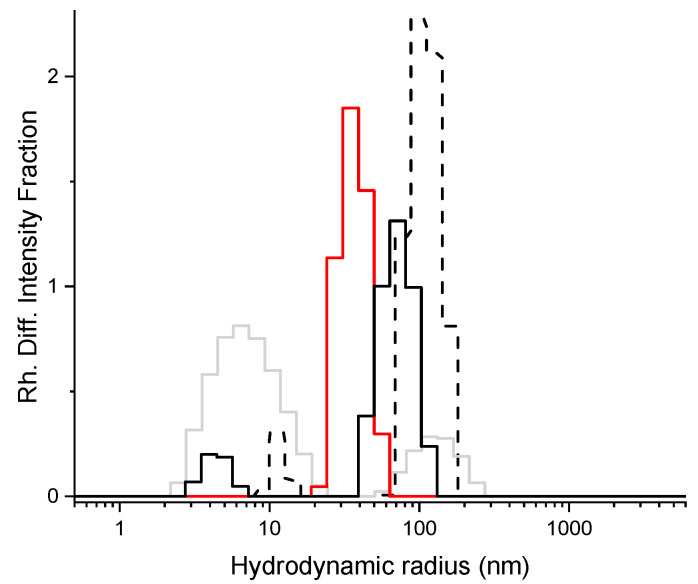
Dynamic Light Scattering (DLS) measurements of GO (black dashed line), PVA (grey line), PVA@GO (black line), and PVA@rGO-Ag1 (red line) in water (samples concentration 12.5 μg/mL).

The size of nanosystems in water dispersion were investigated by Dynamic Light Scattering (DLS) analyses. Generally, DLS values are not indicative of the morphology and size of the graphene nanosystems, but provide information about their hydrodynamic size [63,64]. The functionalization of GO with PVA resulted in two hydrodynamic radius distributions (Figure 6, black line) centered at 5 nm and 70 nm: those are similar to that of the pure PVA (grey line), even while having different relative intensities.

The microwave-assisted procedure for PVA@rGO-AgX synthesis slightly affects the hydrodynamic radius of the nanohybrid system (about 35 nm) as evidenced by PVA@rGO-Ag1 (Figure 6, red line), suggesting an increase of hydrophilicity due to the silver loading.

### 3.3. Preliminary Antibacterial Activity Assay of the PVA@rGO-Ag

In order to investigate the antibacterial performances of the PVA@rGO-Ag nanosystem, a qualitatively empirical test has been performed considering the typical bacterial population of common hand-contacted surfaces.

**Figure 7:**
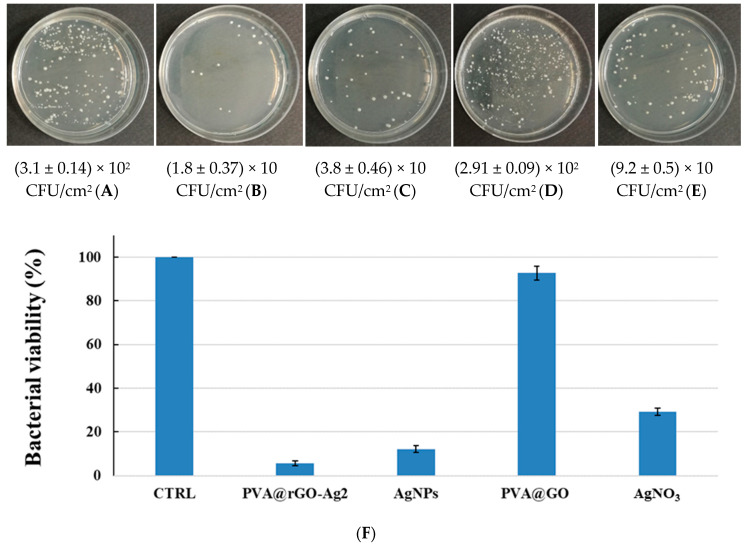
Antibacterial assay in plate count agar after 24 h of incubation: (**A**) Control experiment (CTRL); (**B**) PVA@rGO-Ag2; (**C**) AgNPs; (**D**) PVA@GO; (**E**) AgNO_3_; (**F**) viability percentage. The deviation standard in CFU/cm^2^ has been derived from triplicate test. The CFU for cm^2^ of sampling surface (CFU/cm^2^) values were calculated from Equation (1).

PVA@rGO-Ag2 nanosystem was selected due to the highest AgNPs content because the PVA@rGO-Ag3 does not show clear physicochemical properties of AgNPs (i.e., LSPR signal). The PVA@rGO-Ag2 antibacterial efficacy has been qualitatively compared with the bare AgNPs (the synthetic procedure is described in Materials and Methods), silver nitrate, and PVA@GO (Figure 7).

Control experiment (Figure 7A) and PVA@GO (Figure 7D) Petri dish show a higher bacterial load, in terms of CFU/cm^2^, than all other conditions. As expected, several colony morphologies, such as golden-yellow colonies, large, and white-raised colonies were detectable from cultures in PCA, suggesting the presence of multiple bacterial species in the sampling. Moreover, bacterial viability suggests that the effects of bare PVA@GO towards bacterial proliferation resulted to be negligible (Figure 7F). The condition with AgNO_3_ (Figure 7E) shows a significant reduction of the bacterial load (estimated around 70%, Figure 7F), even if the presence of multiple bacterial species is still maintained. Finally, the conditions with PVA@rGO-Ag2 (Figure 7B) and AgNPs (Figure 7C) show a reduction of 88% and 94% (Figure 7F), with total killing of some bacterial species. The same experiment involving PVA@rGO-Ag1 was performed, but the bacterial viability was not significantly reduced (Figure S6).

## 4. Discussion

Nanotechnologies could fulfil the need to find an alternative to the common organic molecular systems used to make antimicrobial surfaces. The use of AgNPs embedded within a carbon-based nanosystem could provide a combined effect towards bacterial colonization [40]. Indeed, graphene toxicity provides mechanical and oxidative stresses, while the AgNPs are a well-known broad-spectrum antibacterial agent. Moreover, the polymer functionalization is fundamental to ensure a strong sticking towards a substrate and/or a homogeneous mix within a polymer matrix, thanks to the similar chemical–physical features with target substrates.

On this basis, the new NanoHybrid system composed of Polymer, Graphene, and AgNPs—namely, NanoHy-GPS—was designed and has here been systematically described, employing PVA moiety as a polymer model.

Aiming to develop an interchangeable GO-based system, the GO grafting-to procedure has been chosen to allow the use of any polymer having functionalities (as end- and/or side- groups) suitable for esterification reactions. Thanks to the interchangeability of the polymer moiety, the NanoHy-GPS system represents a new concept of on-demand potentially tunable antimicrobial nanosystem.

The synthesis of PVA@GO was performed through a slightly modified procedure (Scheme 1) than that proposed by Salavagione et al. [48], and the successful of the reaction was confirmed through NMR analyses (^1^H-NMR and ^1^H-^1^H COSY, Figures S1 and S2).

Then, the PVA@GO was used as a platform to be decorated with silver nanoparticles (Scheme 1). With this aim, a one-pot microwave-assisted reduction procedure (simultaneously reducing the GO and the silver ions) has been performed. Three so-called PVA@rGO-AgX nanosystems were produced with increasing Ag contents (PVA@rGO-Ag1, -2, and -3) to investigate the microwave-assisted synthesis efficiency and the antibacterial activity.

The presence of the PVA@GO platform influences the AgNPs formation—probably, the different chemica–physical nature of the nucleation sites ensured by the polymer-functionalized carbon platform, coupled with the role of PVA moiety to act as NPs stabilizer, allowed the growth of AgNPs, as revealed by UV-vis measurements (see Figure 1). As a confirmation, in absence of PVA@GO during the microwave-assisted procedure, the AgNPs formation is hindered (as revealed by the absence of LSPR signal, see Figure S4). The quantitative determinations of the silver content have been performed by TGA technique (see Figure 2), which is a reliable approach to determine the metal content within hybrid systems [57,65]. The Ag weight (%) content in the different samples was calculated, obtaining 5%, 16%, and 87% (*w/w*) of Ag for PVA@rGO-Ag1, -2, and -3, respectively.

SEM characterization showed the general morphology of the hybrid systems and the AgNPs distribution (see Figure 3), with the smooth and laminar surface of PVA@GO platform and regular and spherical AgNPs. The SEM images displayed that the increase of AgNPs induces an almost complete covering of the PVA@GO substrate and a formation of nanoparticles agglomerates. Conversely, in sample PVA@rGO-Ag1, with lower amount of AgNPs, the nanoparticles are barely visible and may be embedded in the composite (Figure 3C).

EDX analysis showed the decreasing trend of the amount of AgNPs from PVA@rGO-Ag3 to PVA@rGO-Ag1 samples, confirming the trend of the data obtained through TGA. In addition, the presence of any contaminants is excluded.

To better describe the nanohybrid system morphology, the PVA@rGO-Ag1 sample was selected as a model system, because a higher Ag amount (as PVA@rGO-Ag2) would hide the PVA@rGO substrate. The AFM (Figure 4) and HRTEM micrographs (Figure 5) of PVA@rGO-Ag1 evidenced that the nucleation of the AgNPs occurs onto the GO platform and within PVA matrix as well. This data confirmed the exploitation of both the microchemical environment of GO oxygenated groups and the PVA hydroxyl groups interactions with the external surface of silver nanoparticles [66].

Thanks to the polymer-functionalization, the NanoHy-GPS might ensure a long-lasting coating, paving the way to its antibacterial activity expression over time. The NanoHy-GPS is conceptually suitable to be blended within the polymer used in the production of invasive biomedical devices. Moreover, aiming to replace the commonly used antibacterial agents, it could be also sprayed or applied by dipping on any polymer-based surfaces already produced.

With the aim to potentially use the NanoHy-GPS as an antibacterial agent for surfaces of common-use objects, experimental proof of concept was carried out, taking into account the bacterial population of common hand-contacted surfaces. The results revealed that PVA@rGO-Ag2 exhibited improved antibacterial properties than that of bare AgNPs (Figure 7).

Especially in the context of a worldwide spreading pandemic, the features exposed here could also candidate NanoHy-GPS as an antibacterial agent for common-use objects, helping to reduce the spreading and related toxic effects due to the common disinfectants agents [14,67].

Nevertheless, further investigation on antibacterial activity against pathogens involved in various common infections (i.e., Gram (+) and Gram (−) bacterial cells) are in due course, with the aim of optimizing antimicrobial efficacy by tuning the physical–chemical properties of NanoHy-GPS.

## 5. Conclusions

Here, we reported a flexible synthetic strategy for the fabrication of new NanoHybrid systems (NanoHy-GPS) composed of Polymer, Graphene, and AgNPs. It was shown that the synthetic pathway exploits the microwave irradiation for the simultaneous reduction of silver nanoparticles and GO without the use of reductants and surfactants. AgNPs content in NanoHy-GPS (from 5% to 87%) is quali- quantitatively influenced by the amount of Ag salt used during the microwave-assisted reaction, determining variations in both NPs distribution and size of their agglomerates. The method allows binding of different polymers having suitable active groups (alcohol or amine) and control of the AgNPs content in the nanosystem, giving the capability to tune the interfacial interactions towards targeted substrates (matching their relative chemical nature) and optimizing the homogeneity of the dispersion of the GO-derivatives within specific polymer matrices. The NanoHy-GPS is projected to be a valid alternative towards the common antibacterial agents, which incurs to leaks within the environment and/or within organisms’ tissue.

## Supplementary Materials

The following are available online at https://www.mdpi.com/2079-4991/10/11/2269/s1, Figure S1: ^1^H-NMR spectrum of PVA@GO, Figure S2: COSY ^1^H-NMR spectrum of PVA@GO, Figure S3: Normalized UV-vis spectra of GO and rGO, Figure S4: UV-vis spectra of the AgNO_3_ solutions before and after the microwave-assisted procedure, Figure S5: AFM profiles and height calculations of PVA@rGO-Ag1, Figure S6: Antibacterial assay of PVA@rGO-Ag1.

## References

[1] Kamal G.D., Pfaller M.A., Rempe L.E., Jebson P.J.R. (1991). Reduced Intravascular Catheter Infection by Antibiotic Bonding. JAMA.

[2] Bowersock T.L., Woodyard L., Hamilton A.J., DeFord J.A. (1994). Inhibition of Staphylococci by vancomycin absorbed on triidodecylmethyl ammonium chloride-coated intravenous catheter. J. Control. Release.

[3] Shrivastava A. (2018). Additives for Plastics. Introduction to Plastics Engineering.

[4] McKeen L. (2018). Introduction to Plastics and Polymers. The Effect of Sterilization Methods on Plastics and Elastomers.

[5] Costerton J.W. (1999). Bacterial Biofilms: A Common Cause of Persistent Infections. Science.

[6] Kingshott P., Wei J., Bagge-Ravn D., Gadegaard N., Gram L. (2003). Covalent Attachment of Poly(ethylene glycol) to Surfaces, Critical for Reducing Bacterial Adhesion. Langmuir.

[7] Davies D.G., Chakrabarty A.M., Geesey G.G. (1993). Exopolysaccharide Production in Biofilms - Substratum Activation of Alginate Gene-Expression by Pseudomonas-Aeruginosa. Appl. Environ. Microb..

[8] Davies D.G., Geesey G.G. (1995). Regulation of the Alginate Biosynthesis Gene Algc in Pseudomonas-Aeruginosa during Biofilm Development in Continuous-Culture. Appl. Environ. Microb..

[9] Sutherland I.W. (2001). Biofilm exopolysaccharides: A strong and sticky framework. Microbiology.

[10] Stoodley P., Sauer K., Davies D.G., Costerton J.W. (2002). Biofilms as Complex Differentiated Communities. Annu. Rev. Microbiol..

[11] Costerton J.W., Cheng K.J., Geesey G.G., Ladd T.I., Nickel J.C., Dasgupta M., Marrie T.J. (1987). Bacterial Biofilms in Nature and Disease. Annu. Rev. Microbiol..

[12] Brown M.R.W., Gilbert P. (1993). Sensitivity of biofilms to antimicrobial agents. J. Appl. Bacteriol..

[13] Vasickova P., Pavlik I., Verani M., Carducci A. (2010). Issues Concerning Survival of Viruses on Surfaces. Food Environ. Virol..

[14] Nabi G., Wang Y., Hao Y., Khan S., Wu Y., Li D. (2020). Massive use of disinfectants against COVID-19 poses potential risks to urban wildlife. Environ. Res..

[15] Balmer M.E., Poiger T., Droz C., Romanin K., Bergqvist P.A., Muller M.D., Buser H.R. (2004). Occurrence of methyl triclosan, a transformation product of the bactericide triclosan, in fish from various lakes in Switzerland. Env. Sci. Technol..

[16] Macherius A., Lapen D.R., Reemtsma T., Römbke J., Topp E., Coors A. (2014). Triclocarban, triclosan and its transformation product methyl triclosan in native earthworm species four years after a commercial-scale biosolids application. Sci. Total Environ..

[17] Fang J.-L., Stingley R.L., Beland F.A., Harrouk W., Lumpkins D.L., Howard P. (2010). Occurrence, Efficacy, Metabolism, and Toxicity of Triclosan. J. Environ. Sci. Health Part C.

[18] Weatherly L.M., Gosse J.A. (2017). Triclosan exposure, transformation, and human health effects. J. Toxicol. Environ. Health Part B.

[19] Banerjee I., Pangule R.C., Kane R.S. (2011). Antifouling Coatings: Recent Developments in the Design of Surfaces That Prevent Fouling by Proteins, Bacteria, and Marine Organisms. Adv. Mater..

[20] Kumar A., Vemula P.K., Ajayan P.M., John G. (2008). Silver-nanoparticle-embedded antimicrobial paints based on vegetable oil. Nat. Mater..

[21] Li W.-R., Xie X.-B., Shi Q.-S., Zeng H.-Y., Ou-Yang Y.-S., Chen Y.-B. (2009). Antibacterial activity and mechanism of silver nanoparticles on Escherichia coli. Appl. Microbiol. Biotechnol..

[22] Morones J.R., Elechiguerra J.L., Camacho A., Holt K., Kouri J.B., Ramírez J.T., Yacaman M.J. (2005). The bactericidal effect of silver nanoparticles. Nanotechnology.

[23] Sharma V.K., Yngard R.A., Lin Y. (2009). Silver nanoparticles: Green synthesis and their antimicrobial activities. Adv. Colloid Interface Sci..

[24] Calabrese G., Petralia S., Franco D., Nocito G., Fabbi C., Forte L., Guglielmino S., Squarzoni S., Traina F., Conoci S. (2021). A new Ag-nanostructured hydroxyapatite porous scaffold: Antibacterial effect and cytotoxicity study. Mater. Sci. Eng. C.

[25] Rai M.K., Deshmukh S.D., Ingle A.P., Gade A.K. (2012). Silver nanoparticles: The powerful nanoweapon against multidrug-resistant bacteria. J. Appl. Microbiol..

[26] McShan D., Ray P.C., Yu H. (2014). Molecular toxicity mechanism of nanosilver. J. Food Drug Anal..

[27] Feng Q.L., Wu J., Chen G.Q., Cui F.Z., Kim T.N., Kim J.O. (2000). A mechanistic study of the antibacterial effect of silver ions onEscherichia coli andStaphylococcus aureus. J. Biomed. Mater. Res..

[28] Matsumura Y., Yoshikata K., Kunisaki S.-I., Tsuchido T. (2003). Mode of Bactericidal Action of Silver Zeolite and Its Comparison with That of Silver Nitrate. Appl. Environ. Microb..

[29] Gupta A., Maynes M., Silver S. (1998). Effects of halides on plasmid-mediated silver resistance in Escherichia coli. Appl. Environ. Microbiol..

[30] Sánchez-López E., Gomes D., Esteruelas G., Bonilla L., Lopez-Machado A.L., Galindo R., Cano A., Espina M., Ettcheto M., Camins A. (2020). Metal-Based Nanoparticles as Antimicrobial Agents: An Overview. Nanomaterials.

[31] Zhang X.-F., Liu Z.-G., Shen W., Gurunathan S. (2016). Silver Nanoparticles: Synthesis, Characterization, Properties, Applications, and Therapeutic Approaches. Int. J. Mol. Sci..

[32] Pulit-Prociak J., Banach M. (2016). Silver nanoparticles–a material of the future…?. Open Chem..

[33] Piperno A., Mazzaglia A., Scala A., Pennisi R., Zagami R., Neri G., Torcasio S.M., Rosmini C., Mineo P.G., Potara M. (2019). Casting Light on Intracellular Tracking of a New Functional Graphene-Based MicroRNA Delivery System by FLIM and Raman Imaging. ACS Appl. Mater. Interfaces.

[34] Neri G., Scala A., Barreca F., Fazio E., Mineo P.G., Mazzaglia A., Grassi G., Piperno A. (2015). Engineering of carbon based nanomaterials by ring-opening reactions of a reactive azlactone graphene platform. Chem. Commun..

[35] Neri G., Micale N., Scala A., Fazio E., Mazzaglia A., Mineo P.G., Montesi M., Panseri S., Tampieri A., Grassi G. (2017). Silibinin-conjugated graphene nanoplatform: Synthesis, characterization and biological evaluation. FlatChem.

[36] Cordaro A., Neri G., Sciortino M.T., Scala A., Piperno A. (2020). Graphene-Based Strategies in Liquid Biopsy and in Viral Diseases Diagnosis. Nanomaterials.

[37] Hu W., Peng C., Luo W., Lv M., Li X., Li D., Huang Q., Fan C. (2010). Graphene-Based Antibacterial Paper. ACS Nano.

[38] Akhavan O., Ghaderi E. (2010). Toxicity of Graphene and Graphene Oxide Nanowalls Against Bacteria. ACS Nano.

[39] Liu S., Zeng T.H., Hofmann M., Burcombe E., Wei J., Jiang R., Kong J., Chen Y. (2011). Antibacterial Activity of Graphite, Graphite Oxide, Graphene Oxide, and Reduced Graphene Oxide: Membrane and Oxidative Stress. ACS Nano.

[40] Cobos M., De-La-Pinta I., Quindós G., Fernández M.J., Fernández M.D. (2020). Graphene Oxide–Silver Nanoparticle Nanohybrids: Synthesis, Characterization, and Antimicrobial Properties. Nanomaterials.

[41] Szaraniec B., Pielichowska K., Pac E., Menaszek E. (2018). Multifunctional polymer coatings for titanium implants. Mater. Sci. Eng. C.

[42] Tang J., Chen Q., Xu L., Zhang S., Feng L., Cheng L., Xu H., Liu Z., Peng R. (2013). Graphene Oxide–Silver Nanocomposite As a Highly Effective Antibacterial Agent with Species-Specific Mechanisms. ACS Appl. Mater. Interfaces.

[43] Zhao R., Kong W., Sun M., Yang Y., Liu W., Lv M., Song S., Wang L., Song H., Hao R. (2018). Highly Stable Graphene-Based Nanocomposite (GO–PEI–Ag) with Broad-Spectrum, Long-Term Antimicrobial Activity and Antibiofilm Effects. ACS Appl. Mater. Interfaces.

[44] Kellici S., Acord J., Vaughn A., Power N.P., Morgan D.J., Heil T., Facq S.P., Lampronti G.I. (2016). Calixarene Assisted Rapid Synthesis of Silver-Graphene Nanocomposites with Enhanced Antibacterial Activity. ACS Appl. Mater. Interfaces.

[45] Ocsoy I., Paret M.L., Ocsoy M.A., Kunwar S., Chen T., You M., Tan W. (2013). Nanotechnology in Plant Disease Management: DNA-Directed Silver Nanoparticles on Graphene Oxide as an Antibacterial againstXanthomonas perforans. ACS Nano.

[46] De Faria A.F., de Moraes A.C.M., Marcato P.D., Martinez D.S.T., Durán N., Filho A.G.S., Brandelli A., Alves O.L. (2014). Eco-friendly decoration of graphene oxide with biogenic silver nanoparticles: Antibacterial and antibiofilm activity. J. Nanopart. Res..

[47] Hummers W.S., Offeman R.E. (1958). Preparation of Graphitic Oxide. J. Am. Chem. Soc..

[48] Salavagione H.J., Goómez M.N.A., Martiínez G. (2009). Polymeric Modification of Graphene through Esterification of Graphite Oxide and Poly(vinyl alcohol). Macromolecules.

[49] Creighton J.A., Blatchford C.G., Albrecht M.G. (1979). Plasma resonance enhancement of Raman scattering by pyridine adsorbed on silver or gold sol particles of size comparable to the excitation wavelength. J. Chem. Soc. Faraday Trans. 2.

[50] Davidson C.A., Griffith C.J., Peters A.C., Fielding L.M. (1999). Evaluation of two methods for monitoring surface cleanliness—ATP bioluminescence and traditional hygiene swabbing. Luminescence.

[51] Barbera L., De Plano L.M., Franco D., Gattuso G., Guglielmino S.P.P., Lando G., Notti A., Parisi M.F., Pisagatti I. (2018). Antiadhesive and antibacterial properties of pillar[5]arene-based multilayers. Chem. Commun..

[52] Nečas D., Klapetek P. (2012). Gwyddion: An open-source software for SPM data analysis. Open Phys..

[53] Yao S., Li Y., Zhou Z., Yan H. (2015). Graphene oxide-assisted preparation of poly(vinyl alcohol)/carbon nanotube/reduced graphene oxide nanofibers with high carbon content by electrospinning technology. RSC Adv..

[54] Agnihotri S., Mukherji S., Mukherji S. (2014). Size-controlled silver nanoparticles synthesized over the range 5–100 nm using the same protocol and their antibacterial efficacy. RSC Adv..

[55] Lin J.-J., Lin W.-C., Dong R.-X., Hsu S.-H. (2012). The cellular responses and antibacterial activities of silver nanoparticles stabilized by different polymers. Nanotechnology.

[56] Iqbal M., Zafar H., Mahmood A., Niazi M.B.K., Aslam M.W. (2020). Starch-Capped Silver Nanoparticles Impregnated into Propylamine-Substituted PVA Films with Improved Antibacterial and Mechanical Properties for Wound-Bandage Applications. Polymers.

[57] Mineo P., Abbadessa A., Mazzaglia A., Gulino A., Villari V., Micali N., Millesi S., Satriano C., Scamporrino E. (2017). Gold nanoparticles functionalized with PEGylate uncharged porphyrins. Dye. Pigment..

[58] Neri G., Fazio E., Mineo P.G., Scala A., Piperno A. (2019). SERS Sensing Properties of New Graphene/Gold Nanocomposite. Nanomaterials.

[59] Ajitha B., Kumar Reddy Y.A., Reddy P.S., Jeon H.-J., Ahn C.W. (2016). Role of capping agents in controlling silver nanoparticles size, antibacterial activity and potential application as optical hydrogen peroxide sensor. RSC Adv..

[60] Saha S.K., Chowdhury P., Saini P., Babu S.P.S. (2014). Ultrasound assisted green synthesis of poly(vinyl alcohol) capped silver nanoparticles for the study of its antifilarial efficacy. Appl. Surf. Sci..

[61] Ananth A.N., Daniel S.C.G.K., Sironmani T.A., Umapathi S. (2011). PVA and BSA stabilized silver nanoparticles based surface–enhanced plasmon resonance probes for protein detection. Colloids Surf. B Biointerfaces.

[62] Filippo E., Serra A., Manno D. (2009). Poly(vinyl alcohol) capped silver nanoparticles as localized surface plasmon resonance-based hydrogen peroxide sensor. Sens. Actuators B Chem..

[63] Caccamo D., Currò M., Ientile R., Verderio E.A.M., Scala A., Mazzaglia A., Pennisi R., Musarra-Pizzo M., Zagami R., Neri G. (2020). Intracellular Fate and Impact on Gene Expression of Doxorubicin/Cyclodextrin-Graphene Nanomaterials at Sub-Toxic Concentration. Int. J. Mol. Sci..

[64] Choudhary P., Parandhaman T., Ramalingam B., Duraipandy N., Kiran M.S., Das S.K. (2017). Fabrication of Nontoxic Reduced Graphene Oxide Protein Nanoframework as Sustained Antimicrobial Coating for Biomedical Application. ACS Appl. Mater. Interfaces.

[65] Kumara C., Luo H., Leonard D.N., Meyer H.M., Qu J. (2017). Organic-Modified Silver Nanoparticles as Lubricant Additives. Acs Appl. Mater. Interfaces.

[66] Kyrychenko A., Pasko D.A., Kalugin O.N. (2017). Poly(vinyl alcohol) as a water protecting agent for silver nanoparticles: The role of polymer size and structure. Phys. Chem. Chem. Phys..

[67] Chang A., Schnall A.H., Law R., Bronstein A.C., Marraffa J.M., Spiller H.A., Hays H.L., Funk A.R., Mercurio-Zappala M., Calello D.P. (2020). Cleaning and Disinfectant Chemical Exposures and Temporal Associations with COVID-19—National Poison Data System, United States, 1 January 2020–31 March 2020. Mmwr. Morb. Mortal. Wkly. Rep..

