# Supplementary material for: Polymer-Based Graphene Derivatives and Microwave-Assisted Silver Nanoparticles Decoration as a Potential Antibacterial Agent
Source: Nanomaterials (Basel). 2020 Nov 16;10(11):2269. doi: 10.3390/nano10112269 (PMC7697993; doi:10.3390/nano10112269)
Supplement: Supplementary file 1 [file nanomaterials-10-02269-s001.pdf]

nanomaterials

Supplementary Materials

Polymer-Based Graphene Derivatives and Microwave-Assisted Silver Nanoparticles Decoration as a Potential Antibacterial Agent

Angelo Nicosia [1,*], Fabiana Vento [1], Anna Lucia Pellegrino [1], Vaclav Ranc [2], Anna Piperno [3], Antonino Mazzaglia [4] and Placido Mineo [1,5,6,*]

[1] Department of Chemical Sciences, University of Catania, V.le A. Doria 6, 95125 Catania, Italy; fabiana.vento@phd.unict.it (F.V.); annalucia.pellegrino@unict.it (A.L.P.)
[2] Regional Centre of Advanced Technologies and Materials, Palacký University Olomouc, Šlechtitelů 11, 78371 Olomouc, Czech Republic; vaclav.ranc@upol.cz
[3] Department of Chemical, Biological, Pharmaceutical and Environmental Sciences, University of Messina, V.le F. Stagno d'Alcontres 31, 98166 Messina, Italy; apiperno@unime.it
[4] CNR-ISMN, Istituto per lo Studio dei Materiali Nanostrutturati, V. le F. Stagno d'Alcontres 31, 98166 Messina, Italy; antonino.mazzaglia@cnr.it
[5] Institute for chemical and physical processes CNR-IPCF, Viale F. Stagno d'Alcontres 37, 98158 Messina, Italy
[6] Institute of Polymers, Composites and Biomaterials CNR-IPCB, Via P. Gaifami 18, 95126 Catania, Italy
* Correspondence: angelo.nicosia@unict.it (A.N.); placido.mineo@unict.it (P.M.)

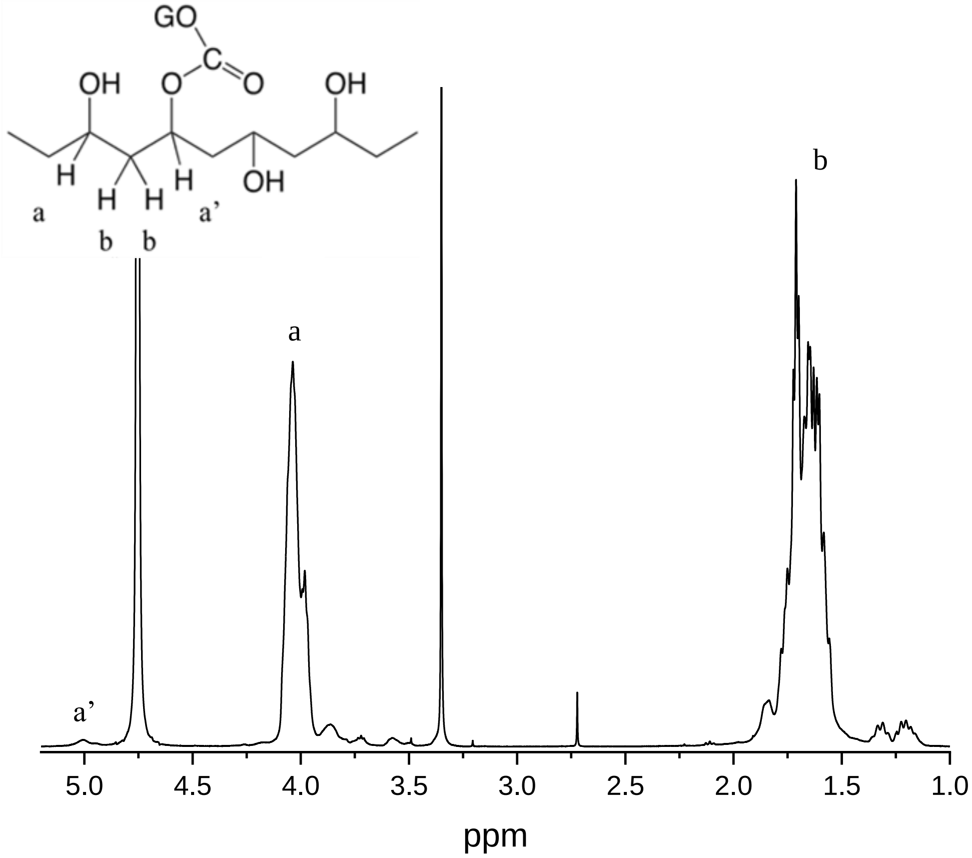

Figure S1. ^{1}H-NMR spectrum of PVA@GO (500 MHz, D_2O), and its structure signals assignment. Methanol residual at 3.35 ppm. DMSO residual at 2.71 ppm. Signals: *b,* 1.15–1.97 ppm (CH_2); *a,* 3.57–4.17 ppm (C–H); *a′*, 5.05 ppm.

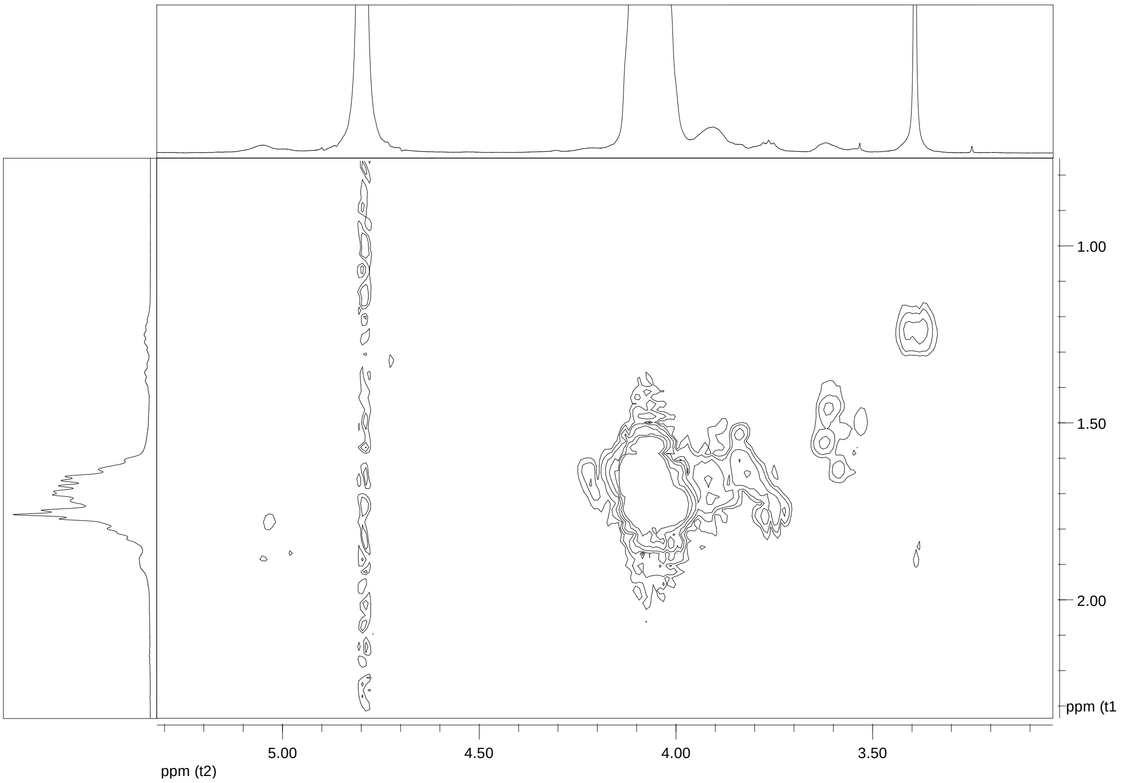

Figure S2. COSY-NMR spectrum of PVA@GO (500 MHz, D_2O).

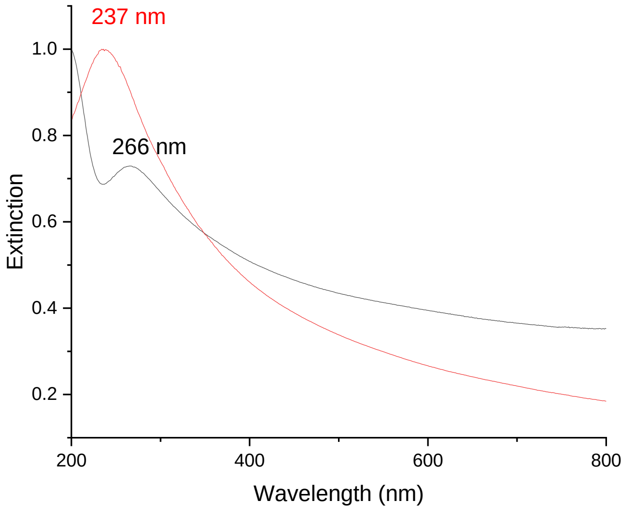

Figure S3. Normalized UV-vis spectra of GO (red line) and rGO (black line) water dispersions (0.3 mg/mL).

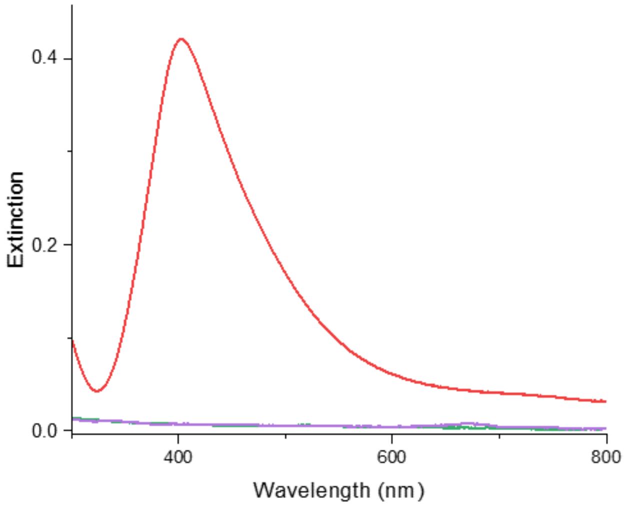

Figure S4. UV-vis spectra of microwave-subjected $AgNO_3$ solution (purple line) and pristine solution (green line), compared with the PVA@rGO-Ag2 (red line).

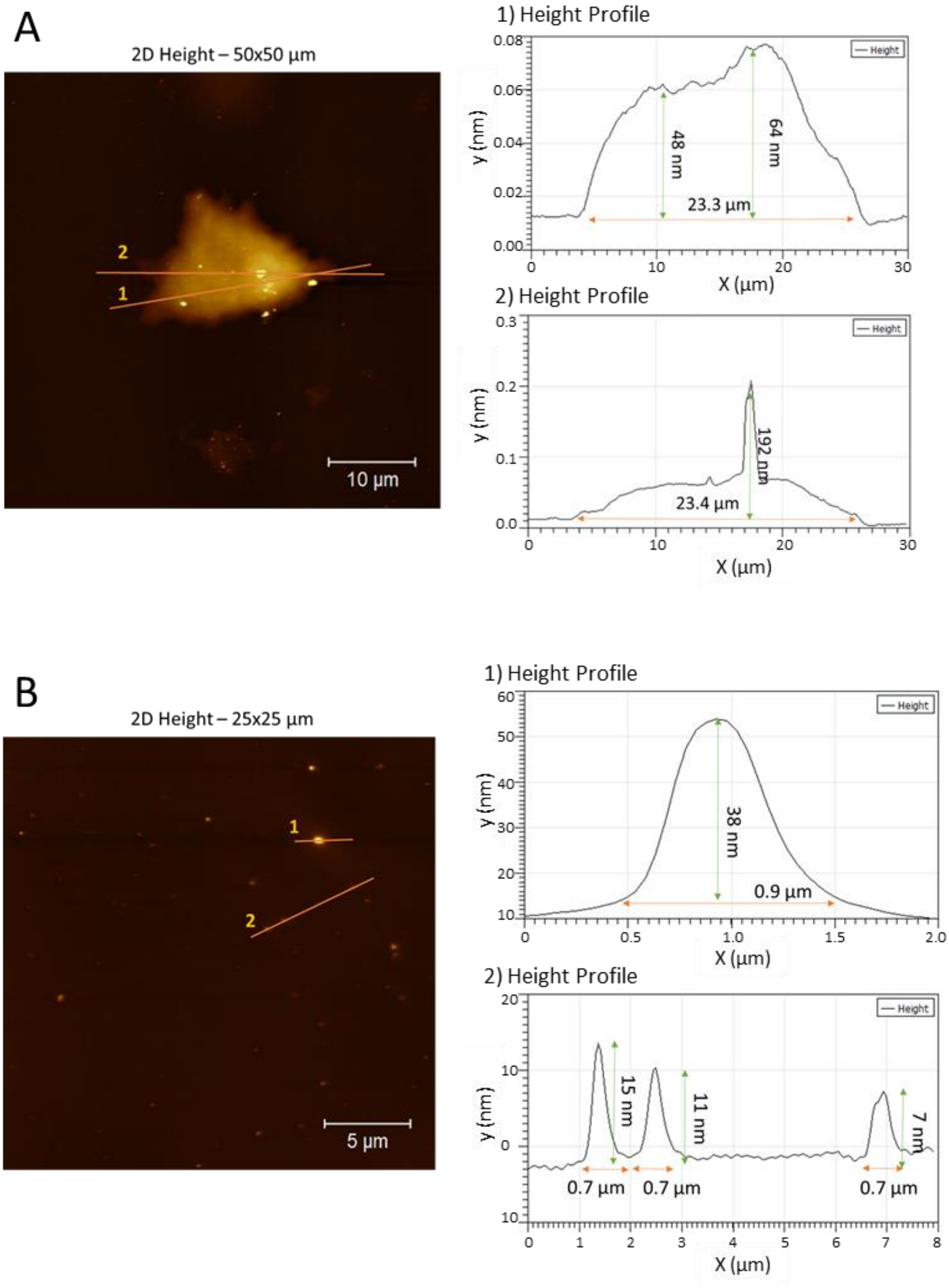

Figure S5. (**A**) AFM topography (50 x 50 µm) of PVA@rGO-Ag1; the height profiles 1 and 2 are reported on the right; (**B**) AFM topography (25 x 25 µm)of PVA@rGO-Ag1; the height profiles 1 and 2 are reported on the right.

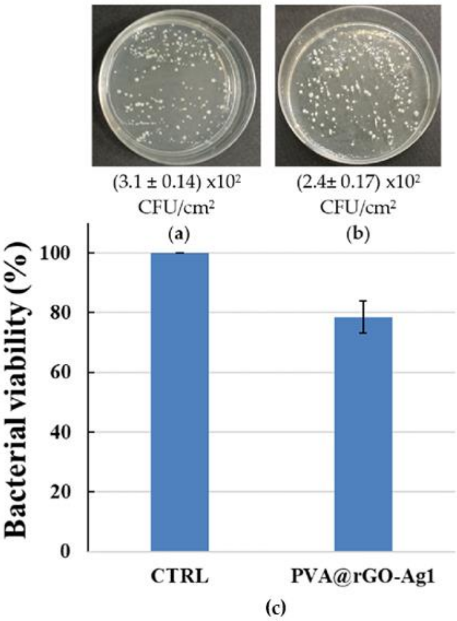

Figure S6. Antibacterial assay in plate count agar after 24 h of incubation: (**a**) control experiment (CTRL); (**b**) PVA@rGO-Ag1; (**c**) Bacterial viability for control and PVA@rGO-Ag1.
